# Supplementary material for: Comparative multiomics analysis of cell physiological state after culture in a basket bioreactor
Source: Sci Rep. 2022 Nov 23;12:20161. doi: 10.1038/s41598-022-24687-4 (PMC9686226; doi:10.1038/s41598-022-24687-4)
Supplement: Supplementary file 1 — Supplementary Information 1. [file 41598_2022_24687_MOESM1_ESM.zip › raw data/Proteomic raw data/3.ProtExprQuantification/PCA.pdf]

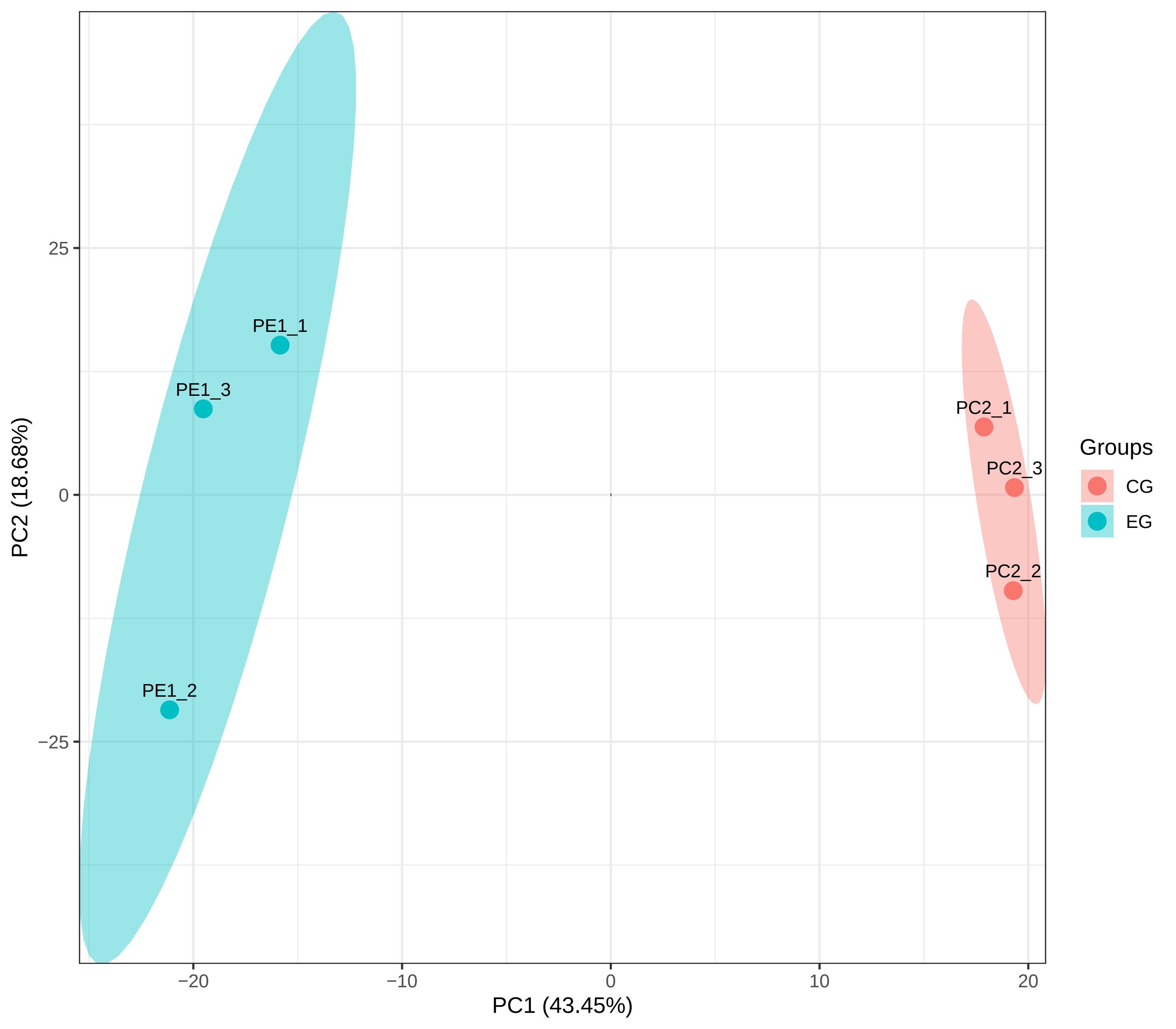
PE1_1
PE1_3
PE1_2
PC2_1
PC2_3
PC2_2
Groups
CG
EG
25
0
−25
−20
−10
0
10
20
PC1 (43.45%)
PC2 (18.68%)
